# Editorial: Neuroinflammation and neurodegeneration from bench to bedside

**DOI:** 10.3389/fneur.2023.1296760

**Published:** 2023-10-06

**Authors:** Wael M. Y. Mohamed, Andrea Kwakowsky

**Affiliations:** ^1^Department of Basic Medical Science, Kulliyyah of Medicine, International Islamic University Malaysia, Kuantan, Pahang, Malaysia; ^2^Pharmacology and Therapeutics, School of Medicine, Galway Neuroscience Centre, University of Galway, Galway, Ireland

**Keywords:** neuroinflammation, neurodegeneration, glia, astrocytes, bedside

The capacity for homeostasis and defense possessed by neuroglia plays a crucial role in neuropathology. In one way or another, glia plays a role in every kind of neurological disorder. Pathological remodeling, atrophy/degeneration, and finally astrogliosis are all stages that occur in injured astrocytes (1). Reactive phenotypes resulting from astrogliosis have been shown to have either neurotoxic or neuroprotective effects (2). Microglia are continually scanning the brain for damage or pathogen-associated molecular patterns (3), and depending on what they find, they may switch between neuroprotective and neurotoxic phenotypes. Oligodendroglia and their progenitors, also known as “NG2 glies,” respond to sickness by proliferating, undergoing Wallerian degeneration, and myelinating (remyelinating) (4). When working together, astrocytes, oligodendrocytes, and neurogranin-2 (NG2-glia), as well as ependymal cells, form macroglia, whereas microglia constitute the CNS's resident phagocytes. Reactive microgliosis and astrogliosis are examples of glial dysfunction that have been associated with alpha-synucleinopathies. Microglial activity has been linked to pathogenic processes in Neurodegenerative Disorders and shown to correlate with disease severity. Microglial activation and gliosis are both very prevalent in the brains of persons with Parkinson's disease. Pathogenic alpha-Syn, a dark pigment found in neurons, and neuromelanin (NM) have been related to microgliosis and Parkinson's disease. NM is only able to elicit a response from catecholaminergic neurons, such as dopaminergic and epinephrinergic neurons (5).

This editorial summarizes the contributions to the Frontiers Research Topic “*Neuroinflammation and neurodegeneration from bench to bedside*.” The articles included three original research papers along with a review article by a total of 16 authors from eight countries. These articles range from a brain imaging study performed on patients for the detection of brain regions responsible for chronic pain in osteoarthritis (Chatterjee et al.) to a study exploring medical records of surgically treated and histologically diagnosed choroid meningioma (CM) patients (Jie et al.) and comprehensive neurological profiling of children and adolescents with Mucopolysaccharidosis type III (MPS III) syndrome (also called Sanfilippo syndrome) (Valle et al.) and critical review of Edible bird's nest (EBN) as a proposed natural supplement with neuroprotective benefits and potential cognitive enhancer (Loh et al.).

Inflammation of the joints in osteoarthritis (OA) is linked to chronic pain. Chatterjee et al. explored the impact of this condition on the brain. Using advanced deep learning (DL) algorithms that examined resting-state functional magnetic resonance imaging (fMRI) data from both OA pain patients and healthy controls identified several new brain regions that had not been identified before, including the occipital lobe, the superior frontal gyrus, the cuneus, the middle occipital gyrus, and the culmen. The authors proposed this tool to be used in OA pain patients for fMRI-based pain recognition, which could lead to enhanced clinical interventions with targeted pain management and improved quality of life of the patients.

Chordoid meningioma (CM) is a rare histologic subtype of meningioma. Jie et al. reviewed the medical records and collected follow-up information on 34 cases who had been surgically treated and histologically diagnosed with CM. The survival analysis demonstrated, that accepting adjuvant radiotherapy and achieving gross total resection correlated with longer progression-free survival for prognosis.

Valle et al. performed a neurological assessment of 10 children and adolescents with MPS III syndrome and reported that neurological, neurobehavioral, and radiological alterations in MPS III patients increased in prevalence and severity with age and were correlated with progressive neurological involvement. MPS III is a lysosomal neurodegenerative disease associated with structural changes, with cortical atrophy detected using MRI in 71% of patients. Behavioral symptoms were reported in seven patients, eight patients developed profound intellectual disabilities and six patients had epilepsy. The findings are important as understanding these neurologic symptoms is critical for better symptom management.

Loh et al. has reviewed and critically discussed the neuroprotective properties of EBN, and its potential as a cognitive enhancer. The composition, antioxidant properties and neuroprotective effects of EBN are outlined. Several mechanisms might be contributing to the cognitive enhancing effects of EBN such as increasing brain-derived neurotrophic factor and sialic acid levels in the hippocampus of offspring in EBN administered women. EBN also increased superoxide dismutase and choline acetyltransferase activities, and lowered acetylcholinesterase activity. In addition, EBNs antioxidant and anti-inflammatory properties might have the ability to improve cognition. Therefore, the supplement might have a potential as a therapeutics for neurodegenerative diseases, but more research is required to provide evidence and explore the mechanism of actions in place.

## Conclusion

In this Research Topic, we focused on the relationship between neuroinflammation and neurodegeneration and its significance. In neurodegenerative diseases, neuronal death is caused by many factors, including network deterioration, impaired synaptic transmission, synaptic loss, and changes in intracellular signaling (6). The development of new therapy targets for neurodegenerative illnesses is extremely challenging due to a lack of understanding of the molecular processes underlying the progression of these diseases (7). There are currently no viable medicines for neurodegenerative illnesses, making this a large unmet medical need. That's why it's so important to discover new ways of treating these diseases and learn more about the mechanisms at play there. More research on the glymphatic system's role in removing proteins prone to intracellular build up is needed to better comprehend neurodegenerative diseases. Neurodegenerative research seems to be influenced by studies of extracellular protein (amyloid-) glymphatic clearance, which in turn inspired interest in the less-studied intracellular protein (tau) and alpha-synuclein. In a manner similar to that of prions, these proteins may hop from one brain cell to the next in connected areas. These findings provide insight on the potential relevance of the glymphatic system in a variety of neurological diseases, including neurodegenerative diseases, by clarifying its role in the clearance of cytotoxic proteins from the central nervous system. The faulty brain clearance mechanisms that lead to the build-up of aberrant proteins in diseases like Alzheimer's and Parkinson's are opening up promising new avenues for diagnosis and treatment of these conditions. Although further study is required to corroborate the concept to alter glymphatic function in these conditions, current research suggests that glymphatic function may be modulated by repurposing already well-described drugs. In addition to its well-known benefits, a healthy lifestyle may also affect the glymphatic system. There is a lot we don't know about the glymphatic system and its function in neurodegenerative diseases, but this field is developing quickly with promising discoveries.

## Author contributions

WM: Conceptualization, Writing—original draft, Writing—review and editing. AK: Conceptualization, Writing—review and editing.
